# Supplementary material for: Cloning and Functional Study of AmGDSL1 in Agropyron mongolicum
Source: Int J Mol Sci. 2024 Aug 30;25(17):9467. doi: 10.3390/ijms25179467 (PMC11395167; doi:10.3390/ijms25179467)
Supplement: Supplementary file 1 [file ijms-25-09467-s001.zip › ijms-3160501-supplementary.pdf]

Supplementary Table S1 Primer sequence

Primer name	Sequence（5′-3′）	Usage
AmGDSL1-q-F	CCAAGAAGCTCGGTTTCGGGAAC	qRT-PCR
AmGDSL1-q-R	GTGCCATTCACCTCCACCTTATCG	
AmGDSL1-F	GGTTCGTCTACAACAACATAGG	RT-PCR validation
AmGDSL1-R	ACGCCATCCCAGCTCACG	
AmGDSL1-c-F	CATGCCATGGGGTTCGTCTACAACAACATAGG	gene cloning
AmGDSL1-c-R	GGACTAGTACGCCATCCCAGCTCACG	
U6-F	GGACATCCGATAAAATTGGAACGATACAG	reference gene
U6-R	AATTTGGACCATTTCTCGATTTATGCGTGT	
